# Effectiveness of Body Roundness Index (BRI) and a Body Shape Index (ABSI) in Predicting Hypertension: A Systematic Review and Meta-Analysis of Observational Studies

**DOI:** 10.3390/ijerph182111607

**Published:** 2021-11-04

**Authors:** Julián F. Calderón-García, Raúl Roncero-Martín, Sergio Rico-Martín, Jorge M. De Nicolás-Jiménez, Fidel López-Espuela, Esperanza Santano-Mogena, Pilar Alfageme-García, Juan F. Sánchez Muñoz-Torrero

**Affiliations:** 1 Department of Nursing, Nursing and Occupational Therapy College, University of Extremadura, 10003 Cáceres, Spain; jfcalgar@unex.es (J.F.C.-G.); rronmar@unex.es (R.R.-M.); fidellopez@unex.es (F.L.-E.); esantano@unex.es (E.S.-M.); 2 Department of Public Health, Centro de Salud Zona Centro de Cáceres, Servicio Extremeño de Salud, 10001 Cáceres, Spain; jorgedenicolas@gmail.com; 3 Department of Nursing, Centro Universitario de Plasencia, 10600 Plasencia, Spain; palfagemeg@unex.es; 4 Department of Internal Medicine, Hospital Universitario de Cáceres, 10003 Cáceres, Spain; juanf.sanchezm@gmail.com

**Keywords:** body roundness index, a body shape index, hypertension, anthropometric indices, systematic review, meta-analysis

## Abstract

Introduction: The body roundness index (BRI) and a body shape index (ABSI) are novel anthropometric indices established to determine both the amount visceral adipose tissue and body fat. Objective: to investigate whether BRI and ABSI are better predictors of hypertension than body mass index (BMI), waist circumference (WC) or waist-to-height ratio (WHtR). *Methods*: A systematic search was conducted in the Scopus, PubMed and Web of Science databases up until 31 December 2020. Results: The estimated pooled area under curve [AUC (95% CI)] for BRI [0.67 (0.65–0.70)] for the prediction of hypertension were superior to that of ABSI (0.58 (0.56–0.60)), similar to that of BMI [0.67 (0.64–0.69)], and lower than those WC [0.68 (0.66–0.70)] and WHtR [0.68 (0.66–0.71)]. Nevertheless, the difference of BRI compared to WC and WHtR in the context of predicting hypertension was non-significant. ABSI was significantly lower (*p* < 0.05) than BRI, BMI, WC and WHtR. Similar findings were observed with the summary receiver operating characteristic curve (AUC-SROC). There were no significant differences between subgroups according to type of population or diagnostic criteria of hypertension. The diagnostic odds ratio (dORs) proved that increased BRI and ABSI were related with an elevated hypertension risk. Conclusions: BRI and ABSI have discriminatory power for hypertension in adult women and men from different populations. Although, WHtR and WC provided the best performance when assessing hypertension, no significant differences were found for BRI. Finally, BRI was significantly better predictor of hypertension than ABSI.

## 1. Introduction

Hypertension is defined as a blood pressure (BP) above the values considered normal damaging to organs such as the heart, brain and kidneys [1]. The global prevalence of hypertension is high [2] and the hypertension treatment is the most common cause for office visits and for the chronic use of prescription medications [3,4]; hypertension is also related with a significant rise in risk of ischaemic stroke, intracerebral haemorrhage, ischaemic heart disease, peripheral artery disease, chronic kidney disease and end-stage renal disease [5,6,7]. The probability of having a cardiovascular event is increased by the elevation in blood pressure [8,9].

Weight gain and obesity are main risk factors for hypertension and are also determinants of the increase in BP [10,11]. Excess weight normally raises BP, and weight loss usually lowers BP [12]. Obesity is primarily related to increased cardiac output and a comparatively normal systemic vascular resistance [13]. Obese subjets have increased activation of the renin-angiotensin-aldosterone system [14]. In addition, numerous mechanisms by which overweight and obesity raise BP have been proposed [15,16].

In most studies, body fat has usually been assessed by a series of anthropometric measurements. Body mass index (BMI) calculated as weight divided by the square of the height (kg/m^2^), is the most widely applied anthropometric measure to define obesity and overweight in clinical and epidemiological studies [17,18,19] and is recommended by World Health Organization (WHO) [20]. Though, this anthropometric index does not distinguish between fat or lean mass, and it does not differentiate the location of central or peripheral fat [21,22]; therefore, waist circumference (WC) and waist-to-hip ratio (WHR) were proposed as indicators of central obesity for their relation with fat distribution [23,24]. The major limitation of WC is that it does not consider the subject’s height and weight [25] and thus may over or underestimate obesity in tall or short individuals [26]. Measurement of the WHR provides no advantage over WC alone and is not recommended as part of the routine obesity evaluation [27], therefore clinicians infrequently use it. Finally, a meta-analysis has revealed that WC, WHR and BMI have equal degrees of association with hypertension [28].

On the other hand, waist-to-height ratio (WHtR) has been proposed as a predictor of metabolic and cardiovascular abnormalities [26] because it addresses the limitations of BMI, WC and WHR. Hence, WHrR is a good indicator to identify hypertension, and in some instances, it is better than WC, WHR and BMI [26,28,29,30]. 

In the last decade, two new anthropometric indices combining traditional measures (height, weight and WC) have been suggested as alternatives to traditional anthropometric indices. In 2012, A Body Shape Index (ABSI) [31], defined as *WC/(BMI^2/3^ × Height^1/2^*), was proposed with the intention of predicting the risk of pathologies that cannot be readily identified by BMI. ABSI has been associated with all-cause mortality, metabolic syndrome, diabetes and hypertension [31,32,33,34]. In 2013, Thomas DM et al. suggested the Body Roundness Index (BRI) [35], defined as *364.2 − 365.5* *× {1 − [(WC/2π)/(0.5* *× height)]^2^}^0.5^*, as a predictor of visceral adiposity tissue and body fat percentage. BRI has proved to be a good predictor of metabolic syndrome in both men and women of diverse nationalities and ethnic groups [34]. BRI was similar to WC and WHtR and superior to BMI, WHR and ABSI. In addition, several observational studies have shown its relationship with hypertension or high BP [36,37,38,39,40,41,42,43,44,45,46,47,48,49]. Observational studies have compared the prediction of hypertension by BRI and ABSI with classical anthropometric indices [36,37,38,39,40,41,42,43,44,45,46,47,48,49], however, there is currently no systematic review and meta-analysis showing whether BRI is a better predictor of hypertension than ABSI, WHtR, WC or BMI. Thus, the aim of this meta-analysis was to determine whether BRI and ABSI are better predictors of hypertension than BMI, WC or WHtR.

## 2. Methods

The method employed in this systematic review and meta-analysis was similar to that used in a previous meta-analysis carried out by our research group [34] according to the Preferred Reporting Items for Systematic Reviews and Meta-Analyses (PRISMA) statement [50].

### 2.1. Search Strategy and Study Selection

A systematic search was conducted in the Web of Science (WOS), Scopus and PubMed databases up until 31 December 2020. The following keywords were used: “Body roundness index” and/or “BRI” and “A body shape index” and/or “ABSI” and “hypertension” or “blood pressure”. All articles with English or Spanish abstracts and full texts were evaluated. No additional filters were applied.

First, two independent reviewers (E.S.-M. and J.M.N.-J.) performed title and abstract screening. Subsequently, the potentially relevant studies were identified for the full-text review. Articles with unclear titles were read entirely. Discrepancies were resolved by agreement with the third reviewer (S.R-M). We used the following inclusion criteria:

(1) Primary observational studies published in peer review journals: cross-sectional or prospective design.
(2) Studies in humans ≥18 years.
(3) Anthropometric indices: BRI and ABSI.
(4) Purpose: to evaluate the predictive value of BRI and ABSI for hypertension or high BP.
(5) For the meta-analysis: studies reporting predictive measures: area under the curve (AUC) with 95% confidence interval (95% CI).

We excluded studies that met any of the following criteria:

(1) Letters to the editor or abstracts from conference proceedings, protocols and review studies.
(2) Studies of adolescents and/or children.
(3) Papers that provided no predictive statistics (AUC 95% CI) for BRI and ABSI for hypertension or high BP.
(4) Articles without an abstract and full text in Spanish or English.

### 2.2. Data Extraction

For each selected article, two reviewers (R.R.-M. and J.F.C.G.) extracted the data, including first author, year and country of publication, study design, sample size (% males), characteristics of the population studied, age range (or median ± SD), follow-up duration (if a longitudinal study), hypertension criteria, adjusted confounders and predictive results (AUC, sensitivity, and specificity) of the anthropometric measures (BRI, ABSI, BMI, WC, and WHtR).

The methodological quality of included studies was assessed according to the Observational Cohort and Cross-sectional Studies from the Heart, Lung and Blood Institute criteria [51] by two independent reviewers (J.F.S.M.T. and F.L.E.). This tool contains 14 criteria scored as 1 if the response was “yes” and 0 if it was “no” or other (i.e., ‘not applicable’, not reported’ or ‘cannot determine’). The scores for each criterion were summed to obtain the total score (ranging from 0 to 14). Discrepancies were resolved by agreement with the third reviewer (S.R.M).

### 2.3. Data Synthesis and Analyses

Articles reporting AUC (95% CI) were included in the meta-analysis. The pooled effect size and their 95% CI for predicting hypertension were calculate for each outcome (BRI, ABSI, BMI, WC, and WHtR) using the inverse variance method. Data for female and male were analysed separately. The data on AUC for each study were pooled using the mean value and standard error (SE) and were weighted by the inverse variance method. SEs were calculated with this measure (SE = upper limit of 95% CI—AUC/1.96). Additionally, a more robust analysis was conducted using studies that published sensitivity and specificity values. We constructed the summary receiver operating characteristic (SROC) curve, which was a measure of the diagnostic accuracy of the anthropometric indices [52,53]. AUC-SROC values were calculated to describe test accuracy. The anthropometric measures were classified in relation to their discriminatory power by the AUC-SROC and AUC and using values suggested by Swets [54], with ≤0.5 deemed to have no discriminatory power, >0.5 to ≤0.7 to have low discriminatory power, >0.7 to ≤0.9 to have good discriminatory power, and 1 to be a perfect test. The sensitivity, specificity, positive likelihood ratio (PLR), negative likelihood ratio (NLR) and diagnostic odds ratio (dOR) along with their corresponding 95% CIs were estimated for the anthropometric indices assessed. We used the DerSimonian and Laird method [55] to calculate pooled estimates of AUC, SROC-AUC and dOR for each included article. Heterogeneity was evaluated using the I^2^ statistic [56], which was interpreted accordingly as follows: modest (0–25%), moderate (25–50%), substantial (50–75%) and considerable (75–100%). We estimated a random-effect model when substantial to considerable heterogeneity was present, and a fixed-effect model was used when there was modest or moderate heterogeneity. The pooled AUC/dOR values of each anthropometric index predicting hypertension were compared by I^2^ statistics and *p*-values. Subgroup analyses were carried out to investigate whether the heterogeneity of articles could be explained by type of population and hypertension criteria. Random-effects meta-regression models were conducted to examine if mean age influenced AUC values. Moreover, sensitivity analyses were carried out to assess the individual influence of each particular article in the pooled AUC by eliminating studies one by one.

We assessed the publication bias by Egger’s test [57]. All analyses were conducted using the Review Manager software (RevMan V.5.3.5, Cochrane Community, London, United Kingdom), Meta-DiSc version 1.4 (Universidad Complutense, Madrid, Spain), and “metagen” and “meta” functions of R version 4.3-2 R Foundation for Statistical Computing, Vienna, Austria). Finally, we considered *p* < 0.05 statistically significant.

## 3. Results

### 3.1. Study Selection 

**Figure 1:**
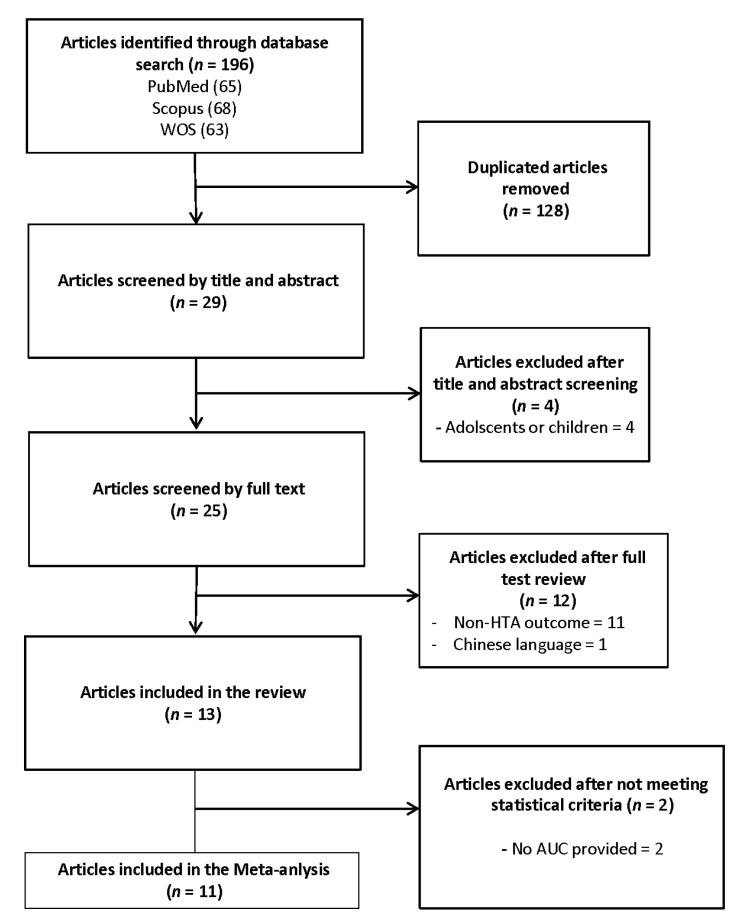
Study selection flow chart.

The systematic search detected 196 references through keyword search, including 63 papers from WOS, 65 from PubMed and 68 from Scopus. Of these, 128 were duplicates, resulting in 29 papers. After titles and abstracts revision, four articles were excluded because they were not conducted on adults. Twenty-five studies were selected for review after full-text evaluation. Of these, 12 papers were excluded. Therefore, 13 articles fulfilled the inclusion criteria and were incorporated in the systematic review, and 11 papers provided sufficient data for the meta-analysis. The study selection process is illustrated in Figure 1.

### 3.2. Study Characteristics

A total of 13 studies were included in this systematic review. Of these, 12 were cross-sectional studies and one was a prospective study with an average follow-up of 2.8 years. All included articles in this systematic review were published between 2016 and 2020. These studies were performed in nine countries including Spain (n = 2), China (n = 5), Nigeria (n = 1), Iran (n = 1), Turkey (n = 1), Peru (n = 1), Republic of Korea (n = 1), and Norway and Poland (n = 1). The number of participants ranged considerably (from 104 to 59,029) between the papers with a median 5225 and an average of 9153. According to population characteristics, 10 studies assessed the general population, and three evaluated populations with specific characteristics (non-obese adults, workers and individuals with daytime hypertension). The minimum age of participants in each included study was ≥18 years. Articles used two different hypertension diagnostic criteria: seven studies established levels of 130/85 mm Hg or antihypertension medication and six established levels of 140/90 mm Hg or antihypertension medication. A total of eight studies adjusted their results for health-related characteristics (e.g., physical activity, diabetes, alcohol intake, smoking status, hypertension and/or others). Basic study characteristics included in the review are shown in the Table 1.

Table 2 presents a summary of the predictive measures employed in the 11 included papers in this meta-analysis. In nine articles, the data were sex-stratified. All studies measured BRI and ABSI, nine also assessed BMI and WC, and eight evaluated WHtR. Predictive measures provided were AUC (95% CI) and OR (95% CI).

### 3.3. Meta-Analysis

We conducted a meta-analysis to examine the performance of BRI and ABSI in predicting hypertension and to investigate whether were superior to BMI, WC and WHtR.

**Figure 2:**
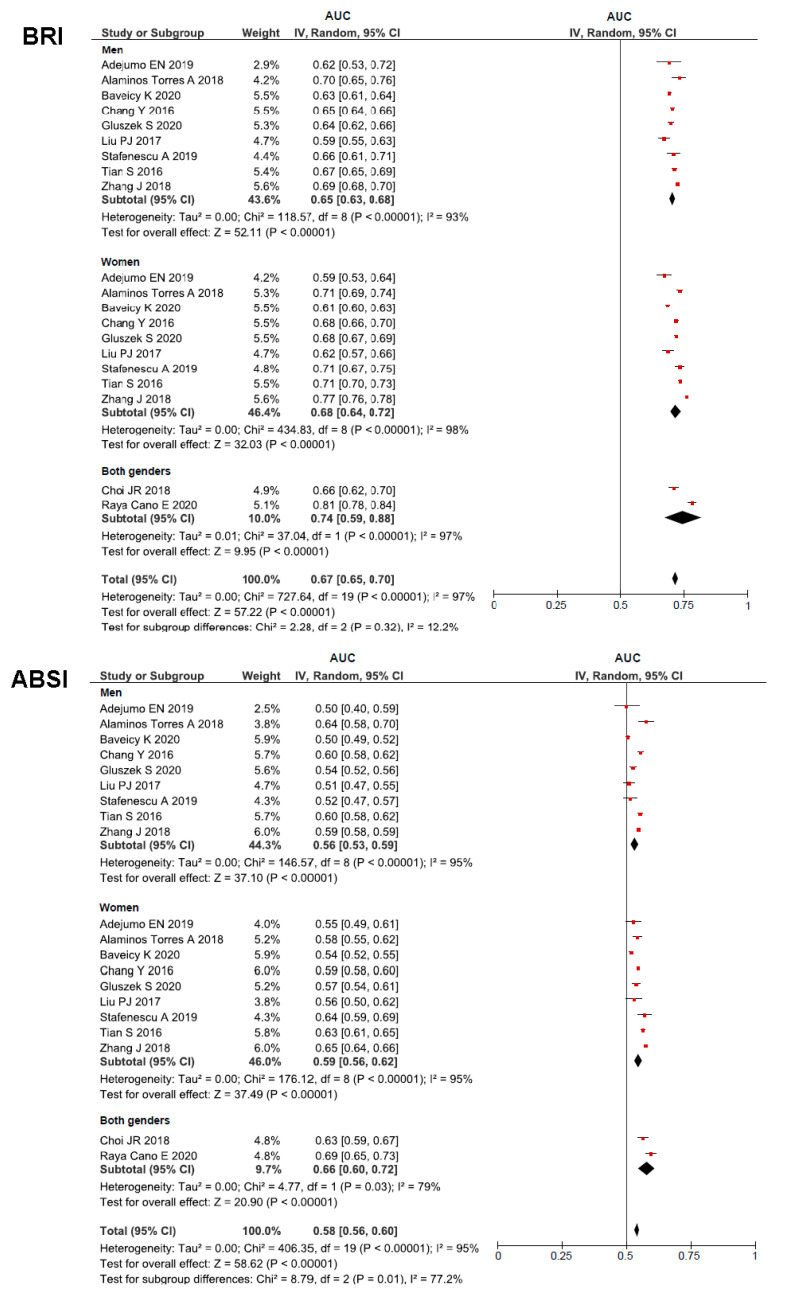

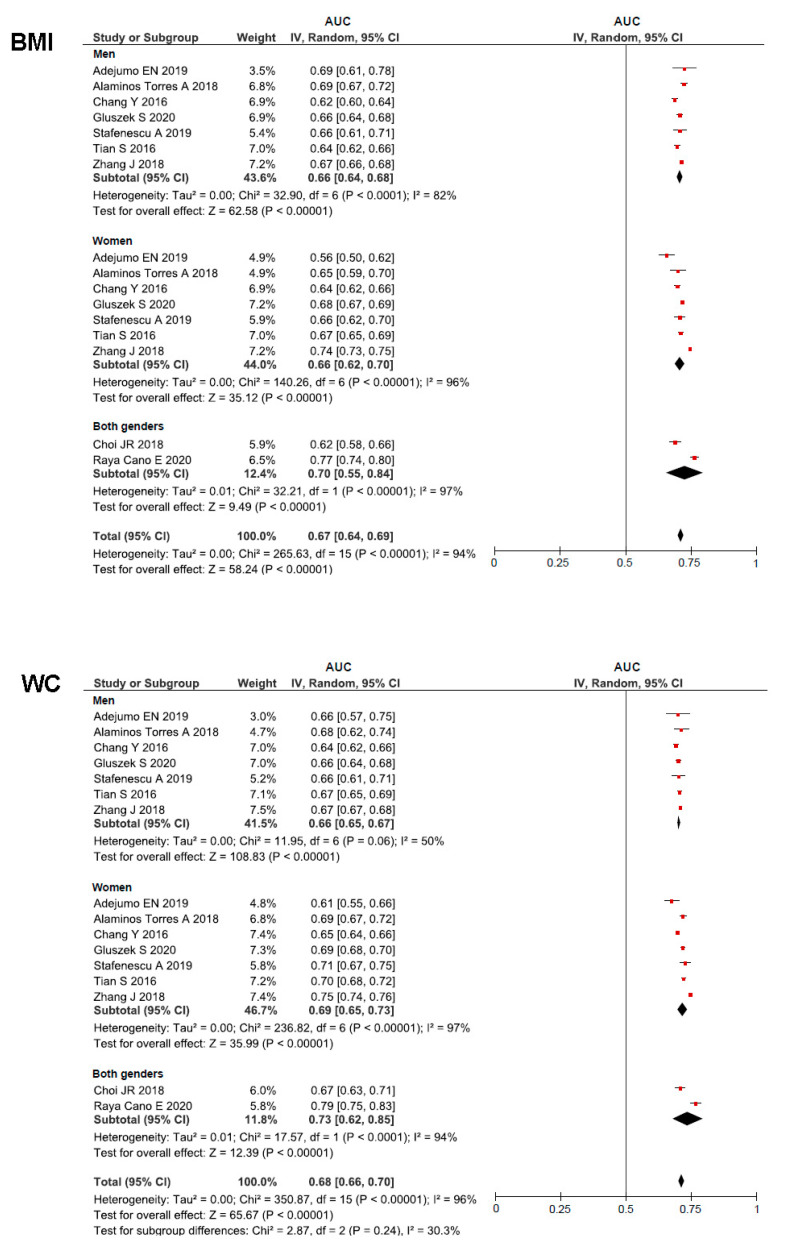

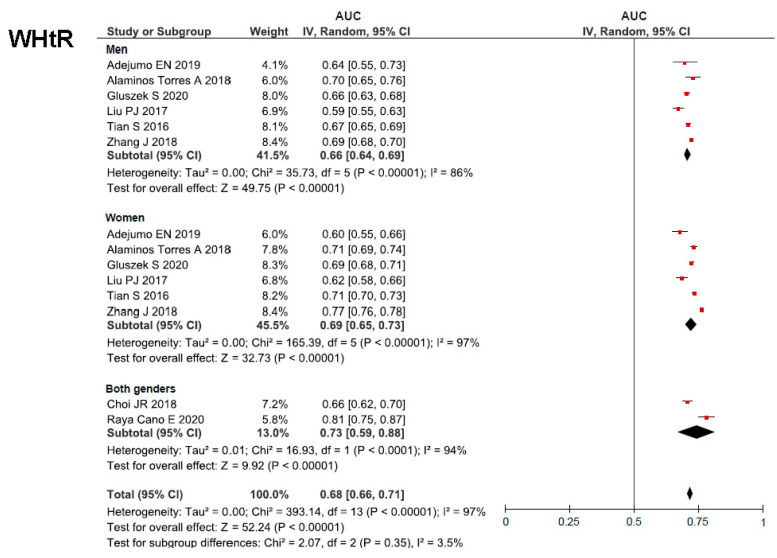
Estimated pooled AUC for BRI, ABSI, BMI, WC, and WHtR.

Figure 2 illustrate the forest plots of the pooled AUC (95% CI) values of BRI, ABSI, BMI, WC and WHtR for hypertension in both men and women. For all anthropometric measure, pooled AUC values were higher than 0.50 and less 0.70 (low discriminatory power). WC and WHtR had greater pooled AUCs (0.66 in men, 0.69 in women and 0.69 in all subjects). Important heterogeneity (I^2^ > 94.0%) across studies was found in all the indices analysed. The estimated pooled AUC for BRI predicting hypertension was 0.09 (0.05–0.13); I^2^ = 96.0%; *p* < 0.001 in men, 0.09 (0.04–0.14); I^2^ = 83.0%; *p* < 0.001 in women and 0.09 (0.05–0.13); I^2^ = 97%; *p* < 0.001 in all subjects and was higher than ABSI. The difference between BRI and BMI, WC and WHtR in predicting hypertension was not significant. Pooled AUCs for ABSI were significantly lower that AUCs for BMI, WC and WHtR in both women and men (I^2^ > 80%; *p* < 0.001). The random-effects meta-regression model (Figures S1–S3) indicated that age entered in the model as a continuous variable was related to the pooled AUCs estimates in men for BMI (β = −0.003; *p* = 0.033) and WC (β = −0.002; *p* = 0.004), but not for BRI, ABSI and WHtR. In women and all subjects, age was not related to any of the anthropometric indices analysed.

The pooled AUC estimates were not significantly modified when individual article data were eliminated from the analysis one at a time (BRI: 0.67 to 0.68; ABSI: 0.58 to 0.59; BMI: 0.66 to 0.67; WC: 0.68 to 0.69; WHtR: 0.67 to 0.70).

The pooled AUC values for each outcome according to type of population and hypertension diagnostic criteria were calculated to examine possible differences (Table 3). Non-significant differences were observed between subgroups for each anthropometric measurement studied.

When we compared pooled AUCs for BRI and ABSI with the rest of anthropometric measures according to type of population and hypertension diagnostic criteria, we found that pooled AUCs for ABSI were significantly lower (I^2^ > 80%; *p* < 0.05) than AUCs for BRI, BMI, WC and WHtR in all subgroups analysed, in both men and women and in all of the subjects, except in European women, where ABSI was not significantly different. However, there were no significant differences between BRI and the other anthropometric measures analysed (BMI, WC and WHtR). 

Only four studies provided specificity and sensitivity values (Table S2). We performed the SROCs to calculate the pooled AUC-SROCs (Figures S4–S6). The AUC-SROC was not determined for BMI and WC for men and women separately because only two papers published the data necessary for estimation. The pooled specificity, sensitivity, NLR, PLR, dOR and AUC-SROCs predicting hypertension are shown in Table 4. The pooled AUC-SROCs (95% CI) for BRI were 0.64 (0.59–0.68) for males, 0.62 (0.52–0.72) for females and 0.64 (0.60–0.69) for all subjects. These values were lower than the pooled AUCs estimated by the inverse variance method. On the other hand, the pooled AUC-SROCs for ABSI were 0.55 (0.49–0.60) for males, 0.59 (0.54–0.65) for females, and 0.57 (0.53–0.61) for all subjects. These values were similar the pooled AUCs estimated by the inverse variance method in women but were inferior in men and all subjects. The pooled AUC-SROCs for BRI were significantly superior to the AUC-SROCs for ABSI in men: 0.09 (0.04–0.014); I^2^ = 85.0%; *p* < 0.01) and all subjects: 0.07 (0.02–0.12); I^2^ = 82.0%; *p* < 0.01). Moreover, AUC-SROCs for ABSI were significantly lower (I^2^ > 80%; *p* < 0.05) than the AUC-SROCs for BMI in all the subjects, WC in all the subjects and WHtR in both sexes and all the subjects. Finally, pooled AUC-SROCs for BRI were non-significantly lower that AUC-SROCs for BMI, WC and WHtR. 

Pooled dORs were calculated for all the anthropometric measures. BMI had pooled dORs greater in men: 2.81 (2.67–2.97), women: 3.99 (2.99–5.31) and all subjects: 3.33 (2.57–4.31). Pooled dORs for ABSI were significantly lower than BRI, BMI and WHtR in men and all the subjects, and WC in all the subjects. However, no significant differences were found between BRI and BMI and WC and WHtR. 

### 3.4. Quality of Studies and Publication Bias

Table S1 shows the assessment results of the quality of the studies included. The mean score was 8.84 out of 14 (range from 8 to 12). No paper scored a 14. Due to the characteristics of the articles included (92% had a cross-sectional design), the lack of sample size justification and repeated evaluation of outcomes during the study period were the most frequent limitations. Egger’s test showed no publication bias (*p* > 0.1).

## 4. Discussion

This systematic review and meta-analysis revealed that BRI, and to a lesser extent ABSI, had discriminatory power for hypertension in adult women and men from different populations. The estimated pooled AUCs for BRI predicting hypertension were greater than for ABSI, similar to BMI and lower than WC and WHtR. Nevertheless, the differences between BRI and BMI, WHtR and WC in predicting hypertension were non-significant. The estimated pooled AUCs for ABSI predicting hypertension were significantly lower than the other anthropometric indices analysed. The pooled AUC-SROCs for BRI were not significantly lower than the AUC-SROCs for BMI, WC and BMI but were significantly higher than the AUC-SROCs for ABSI. All the anthropometric indices analysed had significantly higher AUC-SROCs than ABSI. Finally, pooled dORs showed that higher BRI, ABSI and the other anthropometric indices analysed were related with raised hypertension risk.

There is well-established evidence that overweight and obesity are related to augmented risk for hypertension [16,58]. Excess weight usually increases BP, and weight loss generally lowers BP [12,59]. The raise of the risk of hypertension, overweight and obesity increases cardiovascular risk through adverse effects on lipids, insulin resistance, and other cardiometabolic processes, therefore, weight reduction is recommended in hypertensive patients with overweight or obesity for control of metabolic risk factors [12,58]. The degree of the effect of behavioural weight loss on BP has been examined previously in a meta-analysis of eight clinical trials that involved a total of 2100 hypertensive patients [60], where the mean reduction in systolic/diastolic BP was 4.5/3.2 mmHg. Increased adiposity, whether assessed as higher BMI [10,26,40,61,62] or larger WC [26,40,63], was strongly associated with greater BP and development of hypertension. Recently, a meta-analysis [29] of more than 2.3 million individuals has observed a relative risk (RR) of developing hypertension of 1.49 (1.41–1.58) for a 5 kg/m^2^ increment in BMI (49%) and 1.27 (1.15–1.39) for a 10 cm increment in WC (27%). Although the ideal BMI is not clear, maintenance of a BMI of approximately 20–25 kg/m^2^ and WC < 88 cm for women and <102 cm for men is suggested for hypertensive patients to reduce BP and non-hypertensive individuals to prevent hypertension [58]. The main limitation of BMI is that it is not able to differentiate between fat and lean mass, and it does not discriminate between central or peripheral adiposity [21,22]; in addition, there is evidence that decreased muscle mass and increased fat mass is related with an augmented risk of early death [64]. On the other hand, WC does not consider the individual’s height and weight [25] and can over or underestimate obesity in tall or short subjects [26].

Because of the limitations of BMI and WC, abdominal obesity indices, such as WHR and WHtR, have been explored as better predictors of cardiometabolic abnormalities [30,65]. Both WHR and WHtR have been associated with hypertension and higher BP [26,28,29,66]. A 0.1-unit increment in WHR and WHtR was related with 37% (RR: 1.37 (1.24–1.51)) and 74% (RR: 1.74 (1.35–2.13) higher risk of hypertension, respectively [29]. Currently, the guidelines of the medical societies do not recommend WHR as part of the routine obesity evaluation, because provides it no advantage over WC alone [34,67,68,69]. WHtR <0.5 has been established as a reference value to prevent hypertension and to decrease BP in hypertensive patients [65]. 

Recently, other anthropometric indices combining weight, height, WC and/or hip circumference have been proposed [70]. The BRI was designed to determine both the amount of visceral adipose tissue and body fat using WC in relation to height, which allows estimation of the shape of the human body figure as an oval or ellipse [35]. Several observational studies have revealed that the BRI could be utilized as an adipose indicator to assess the existence of hypertension or high BP [36,37,38,39,40,41,42,43,44,45,46,47,48,49]. On the other hand, ABSI is the most researched anthropometric index so far [31,71] and is based on WC adjusted for height and weight. The objective of the ABSI is to determine disease risks that are not detected by BMI [31]. A previous meta-analysis showed that an elevation of one standard deviation in ABSI was related with a 13% higher hypertension risk.

This is the first meta-analysis, including data on more than 118,000 subjects, which analyses the scientific research according to the BRI’s performance to predict hypertension. However, several meta-analyses in adult populations have been published for BMI, WC and WHtR [26,28,29,30,66] and one for ABSI [32]. The members of The Obesity Asia Collaboration published the first meta-analysis that compared BMI’s performance against WC, WHR and WHtR in the discrimination of hypertension [66]. That study showed that pooled AUCs for WHtR were higher that WHR, WC and BMI in both males and females in all of the regions studied. Later, Lee et al. [30] concluded that statistical evidence supports the advantage of measures of centralised obesity, especially WHtR, over BMI, for identifying cardiovascular risk factors in both women and men. For hypertension, pooled AUCs for WHtR were greater than for BMI, WC and WHtR. Statistical comparison of the pooled AUCs showed that only WHtR (in males) was weakly, though significantly, superior at predicting hypertension against BMI (0.64 vs. 0.68; *p* = 0.04). In 2012, Ashwell et al. [26] evaluated the discriminative power of WC, WHtR and BMI to distinguish cardiovascular risk factors. For hypertension, pooled AUCs for WHtR were higher than for WC and BMI. Among women in 19 study groups, significant differences were not found. Conversely, among men (18 study groups), WHtR was significantly greater than BMI (0.69 vs. 0.65; *p* = 0.047). The most recent meta-analysis have published similar results [28], where WHtR was confirmed as a reliable predictor to identifying at augmented risk of hypertension in those subjects, and in some instances, it was better than WC, WHR and BMI. The results of the meta-analysis carried out by Jayedi et al. [29] reported an RR for the development of hypertension of 74% for every 0.1-unit increment in WHtR, 49% and 16% for 5 kg/m^2^ and every 1 kg/m^2^ increment in BMI, respectively, 27% for a 10 cm increment in WC and 37% for every 0.1-unit increment in WHR. According to the ABSI, Ji et al. [32] used its performance in determining type 2 diabetes, hypertension cardiovascular disease and all-cause mortality and compared the differential prediction between ABSI with BMI and WC. Meta-analysis showed that a one standard deviation rise in ABSI was related with a rise in the odds of hypertension of 13% and the estimated pooled AUCs for ABSI in predicting hypertension were 0.58 (0.54, 0.62) I^2^ = 97.1%). The estimated increase in hypertension risk associated with a one standard deviation increase in ABSI was 29% lower than that related with a one standard deviation increase in BMI and WC. The estimated pooled AUC for ABSI in predicting hypertension was found to be 0.03 (0.01, 0.06; I^2^ = 79.0%) and 0.04 (0.01, 0.07; I^2^ = 95.0%) lower than that of BMI and WC, respectively. No meta-analysis indicated whether BRI is a superior indicator or of hypertension than ABSI, WHtR, WC or BMI. In our study, the estimated pooled AUCs for BRI predicting hypertension were greater than for ABSI, similar to BMI and lower than WHtR and WC. However, the differences between BRI and BMI, WHtR and WC in predicting hypertension were non-significant. The estimated pooled AUCs for ABSI predicting hypertension were significantly lower than the other anthropometric indices analysed. WC and WHtR had greater pooled AUCs. On the other hand, the pooled AUC-SROCs for BRI were not significantly lower than AUC-SROCs for BMI, WC and BMI but were significantly greater than AUC-SROCs for ABSI. All the anthropometric indices analysed had significantly higher AUC-SROCs than ABSI. BMI had the greatest pooled AUC-SROCs for all of the subjects and WHtR did when we studied men and women separately. In our analysis, we did not include WHR because only four studies reported on its measurement [37,39,40,45]. 

Obesity and weight gain are major risk factors for hypertension and are also causes of the rise in blood pressure that is commonly observed with ageing [10,11]. In our study, the random-effects meta-regression model did not indicate that age was related to the pooled AUC estimates in the anthropometric indices studied, except for BMI and WC in men. 

It is known that the cut-off points of anthropometric indices based on non-Asian populations are not applicable to Asians [72]. Ethnicity is a significant modifier in the relationship between cardiovascular risk factors and simple anthropometric measures, which is applicable to both women and men [73]. In this meta-analysis, the pooled AUC values for anthropometric indices according to type of population and diagnostic criteria of hypertension were assessed. There were no significant differences between subgroups for each anthropometric indices studied; however, members of the Obesity in Asia Collaboration reported that the association between hypertension and WHtR, WHR, WC and BMI was significantly weaker among non-Asians compared to Asian populations [66]. Furthermore, it has been documented that the presence of cardiovascular risk factors in individuals of Chinese origin appears with lower WC and BMI values than in European individuals [74,75,76]. Currently, no studies have compared ABSI and BRI values between diverse ethnic populations. 

Although BMI, WC and WHtR were better to BRI for detecting the presence of hypertension, no significant differences were found in the pooled AUCs, dORs or AUC-SROCs for predicting hypertension, proposing that BRI could be used as an additional or alternative adiposity measurement in evaluating hypertension. On the other hand, our results suggest that ABSI is a worse anthropometric index for predicting hypertension that BRI, WHtR and traditional anthropometric indices (BMI and WC). 

This systematic review and meta-analysis has several potential limitations. First, some articles were not considered because they were grey literature or were written in languages other than Spanish or English. Second, the inverse variance method is not the most appropriate method for this type of meta-analysis. Currently, the most rigorous and recommended methods are the SROC model by bivariate random effects meta-analysis of specificities and sensitivities and hierarchical ROC (HROC) model [52,77,78]. To resolve this inconvenience, SROC curves were created through studies that provided specificity and sensitivity values. Although the use of bivariate random effects models or HSROC have been suggested [79], Moses’ SROC model achieves similar results [80]. Third, some articles used in this meta-analysis did not provide the AUC of WHtR, BMI and/or WC, and the subsequent pooled AUCs did not include the same number of papers for ABSI and BRI as for the other of the anthropometric indices, so there could be a comparison bias. Similar inconvenience occurred for AUC-SROCs, where specificity and sensitivity for BMI, WC and WHtR were not reported. Fourth, the results showed a substantial or considerable level of heterogeneity, and thus should be interpreted with caution. Finally, all studies included in our meta-analyses were observational, consequently, a cause-effect association cannot be inferred. The major strength of this meta-analysis, including data on more than 118,000 subjects, was to assess the performance of BRI and ABSI in predicting hypertension and to compare it with traditional anthropometric indices (BMI, WC and WHtR). This is the first systematic review and meta-analysis of the hypertension discriminatory power of ABSI and BRI, emphasizing that the oldest of the included articles were reported in 2016 and that most were published in 2019 and 2020 (>60%). 

## 5. Conclusions

In summary, this systematic review and meta-analysis, including data on more than 118,000 subjects, is the first to prove that BRI is a possible predictor and is superior to ABSI in predicting hypertension in adult women and men from different populations. WHtR and WC provide the best performance when assessing hypertension, although no significant differences were found with BRI. In contrast, ABSI was significantly inferior to BRI, BMI, WC and WHtR. Finally, dORs showed that increased BRI and ABSI are related with increased hypertension risk. Future studies should examine the prospective association between novel anthropometric indices (ABSI and BRI) and negative health outcomes in different population and age groups.

## Figures and Tables

**Table 1:** Basic characteristics of the studies included in the review.

Ref.	Author (Year) [Reference]	Country	Study Design	Sample Size (% Male)	Population Chararsterics	Age Range and/or Mean ± SD	Follow up Years	HTA Criteria	Adjustment
1	Adejumo, EN (2019) [36]	Nigeria	Cross-sectional	535 (27.3%)	General population	≥18 47.05 ± 14.34	-	130/85 mm Hg or antihypertensive medication	-
2	Baveicy, K (2020) [37]	Iran	Cross-sectional	8790 (52.2%)	General population	35–65	-	130/85 mm Hg or antihypertensive medication	Age, smoking status (current/former, never), alcohol intake (units per week) and menopause.
3	Candan, S (2020) [38]	Turkey	Cross-sectional	104 (51.9%)	Daytime hypertension	47.6 ± 12.1	-	140/90 mm Hg or antihypertensive medication	-
4	Chang, Y (2016) [39]	China	Cross-sectional	11,345 (46.3%)	General population	≥35	-	140/90 mm Hg or antihypertensive medication	Age, ethnicity, family income, education, physical activity, salt intake, smoking and alcohol status, FPG, and serum lipid.
5	Choi, JR (2018) [40]	Republic of Korea	Prospective cohort	1718 (36.7%)	General population	39–72	2.8	140/90 mm Hg or antihypertensive medication	Age, gender, smoking status, alcohol intake, regular exercise, SBP and total cholesterol at baseline.
6	Głuszek, S (2020) [41]	Polish and Norwegian	Cross-sectional	12,328 (33.2%)	General population	55.7 ± 5.4	-	130/85 mm Hg or antihypertensive medication	-
7	Liu, PJ (2017) [42]	China	Cross-sectional	1596 (44.5%)	Non-obeses adults	20–60	-	High BP = Prehypertension: 120–139/80–89 mm Hg and hypertension; 140/90 mm Hg or antihypertensive medication	-
8	Raya Cano, E (2020) [43]	Spain	Cross-sectional	636 (32.1%)	Workers	45.1 ± 8.8	-	130/85 mm Hg or antihypertensive medication	Age and gender.
9	Stafenescu, A (2019) [44]	Peru	Cross-sectional	1518 (37.3%)	General population	39.3 ± 15.0	-	130/85 mm Hg or antihypertensive medication	Age, smoking status and alcohol
10	Tian, S (2016) [45]	China	Cross-sectional	8126 (46.5%)	General population	18–85	-	140/90 mm Hg or antihypertensive medication	Age, smoking, alcohol status
11	Tian, T (2020) [46]	China	Cross-sectional	8040 (44.9%)	General population	54.7 ± 15.1		130/85 mm Hg or antihypertensive medication	Age, drinking and smoking conditions.
12	Alaminos Torres, A (2019) [47]	Spain	Cross-sectional	5225 (40.2%)	General population	18–75	-	130/85 mm Hg or antihypertensive medication	-
13	Zhang J (2018) [48]	China	Cross-sectional	59,029 (61.2%)	General population	18–80	-	140/90 mm Hg or antihypertensive medication	Age

Abbreviations: BP: Blood Pressure; FPG: Fasting plasma glucose; HTA: Hypertension; SBP: Systolic blood pressure; SD: Standard deviation.

**Table 2:** Measures of the studies included in the meta-analysis.

First Author (Year) [Reference]	Outcome Assesment	BRI	ABSI	BMI	WC	WHtR
Adejumo, EN (2019) [36]	AUC (95% CI)	Men: 0.624 (0.531–0.717) Women: 0.588 (0.532–0.644)	Men: 0.497 (0.402–0.592) Women: 0.553 (0.495–0.611)	Men: 0.694 (0.607–0.781) Women: 0.557 (0.498–0.615)	Men: 0.656 (0.565–0.747) Women: 0.607 (0.551–0.664)	Men: 0.641 (0.549–0.733) Women: 0.602 (0.546–0.658)
Baveicy, K (2020) [37]	AUC (95% CI)	Men: 0.628 (0.614–0.642) Women: 0.614 (0.599–0.629)	Men: 0.502 (0.487–0.516) Women: 0.537 (0.522–0.552)			
	OR (95% CI)	Men: 2.13 (1.78–2.54) Women: 1.85 (1.58–2.17)	Men: 1.85 (1.58–2.17) Women: 1.24 (1.06–1.46)			
Chang ,Y (2016) [39]	AUC (95% CI)	Men: 0.65 (0.64–0.67) Women: 0.68 (0.67–0.70)	Men: 0.60 (0.58–0.61) Women: 0.59 (0.58–0.61)	Men: 0.62 (0.60–0.63) Women: 0.64 (0.62–0.65)	Men: 0.64 (0.62–0.65) Women: 0.65 (0.64–0.67)	
	OR (95% CI)	Men: Q1: Ref Q4: 3.49 (2.86–4.21) Women: Q1: Ref Q4: 3.06 (2.56–3.67)	Men: Q1: Ref Q4: 1.30 (1.06–1.58) Women: Q1: Ref Q4: 1.19 (1.04–1.34)	Men: Q1: Ref Q4: 2.43 (2.01–2.98) Women: Q1: Ref Q4: 2.10 (1.70–2.62)	Men: Q1: Ref Q4: 3.18 (2.55–3.94) Women: Q1: Ref Q4: 2.68 (2.22–3.23)	
Choi, JR (2018) [40]	AUC (95% CI)	0.662 (0.625–0.700)	0.627 (0.587–0.667)	0.623 (0.582–0.664)	0.672 (0.634–0.711)	0.662(0.625–0.700)
	OR (95% CI)	Q1: Ref Q4: 4.46 (2.39–8.34)	Q1: Ref Q4: 1.72 (0.96–3.08)	Q1: Ref Q4: 3.18 (1.91–5.28)	Q1: Ref Q4: 4.79 (2.49–9.20)	Women: Q1: Ref Q4: 4.51 (2.41–8.43)
Głuszek, S (2020) [41]	AUC (95% CI)	Men: 0.638 (0.616–0.659) Women: 0.681 (0.669–0.693)	Men: 0.542 (0.519–0.565) Women: 0.575 (0.541–0.608)	Men: 0.660 (0.638–0.681) Women: 0.681 (0.668–0.694)	Men: 0.657 (0.636–0.678) Women: 0.691 (0.678–0.704)	Men: 0.655 (0.633–0.676) Women: 0.694 (0.681–0.707)
Liu, PJ (2017) [42]	AUC (95% CI)	Men: 0.587 (0.545–0.629) Women: 0.618 (0.574–0.662)	Men: 0.511 (0.468–0.554) Women: 0.558 (0.497–0.620)			Men: 0.589 (0.547–0.631) Women: 0.619 (0.575–0.663)
Raya Cano, E (2020) [43]	AUC (95% CI)	0.81 (0.78–0.85)	0.69 (0.65–0.74)	0.77 (0.74–0.81)	0.79 (0.75–0.82)	0.81 (0.75–0.85)
Stafenescu, A (2019) [44]	AUC (95% CI)	Men: 0.66 (0.61–0.71) Women: 0.71 (0.67–0.75)	Men: 0.52 (0.47–0.57) Women: 0.64 (0.59–0.68)	Men: 0.66 (0.61–0.71) Women: 0.66 (0.62–0.71)	Men: 0.66 (0.61–0.71) Women: 0.71 (0.67–0.75)	
	OR (95% CI)	Men: 1.41 (1.21–1.66) Women: 1.29 (1.16–1.42)	Men: 0.98 (0.94–1.02) Women: 1.04 (1.01–1.07)	Men: 1.14 (1.08–1.20) Women: 1.09 (1.05–1.13)	Men: 1.05 (1.03–1.07) Women: 1.05 (1.03–1.07)	
Tian, S (2016) [45]	AUC (95% CI)	Men: 0.668 (0.650–0.687) Women: 0.714 (0.698–0.730)	Men: 0.597 (0.578–0.616) Women: 0.628 (0.610–0.646)	Men: 0.639 (0.620–0.658) Women: 0.667 (0.649–0.686)	Men: 0.667 (0.649–0.686) Women: 0.698 (0.681–0.715)	Men: 0.668 (0.650–0.687) Women: 0.714 (0.698–0.730)
	OR (95% CI)	Men: Q1: Ref Q4: 3.87 (3.11–4.82) Women: Q1: Ref Q4: 4.00 (3.11–5.15)	Men: Q1: Ref Q4: 1.48 (1.19–1.83) Women: Q1: Ref Q4: 1.42 (1.13–1.79)	Men: Q1: Ref Q4: 4.53 (3.62–5.65) Women: Q1: Ref Q4: 5.02 (3.97–6.34)	Men: Q1: Ref Q4: 4.67 (3.74–5.83) Women: Q1: Ref Q4: 4.32 (3.38–5.52)	Men: Q1: Ref Q4: 3.87 (3.11–4.82) Women: Q1: Ref Q4: 4.00 (3.11–5.15)
Alaminos Torres, A (2019) [47]	AUC (95% CI)	Men: 0.705 (0.649–0.761) Women: 0.711 (0.686–0.735)	Men: 0.644 (0.583–0.704) Women: 0.583 (0.55–0.611)	Men: 0.692 (0.668–0.716) Women: 0.646 (0.588–0.705)	Men: 0.681 (0.624–0.738) Women: 0.692 (0.667–0.717)	Men: 0.705 (0.649–0.761) Women: 0.711 (0.686–0.735)
Zhang, J (2018) [48]	AUC (95% CI)	Men: 0.690 (0.685–0.695) Women: 0.769 (0.761–0.778)	Men: 0.586 (0.581–0.591) Women: 0.648 (0.638–0.659)	Men: 0.667 (0.662–0.672) Women: 0.738 (0.728–0.748)	Men: 0.673 (0.668–0.678) Women:0.752 (0.743–0.762)	Men: 0.690 (0.685–0.695) Women: 0.769 (0.761–0.778)
	OR (95% CI)	Men: 1.807 (1.756–1.860) Women: 1.646 (1.572–1.723)	Men: 1.073 (1.043–1.104) Women: -	Men: 1.956 (1.899–2.014) Women: 1.930 (1.839–2.026)	Men: 1.837 (1.783–1.892) Women: 1.700 (1.622–1.781)	Men:1.860 (1.805–1.917) Women: 1.721 (1.640–1.807)

Abbreviation: AUC: Area Under Curve; ABSI: A Body Adiposity Index; BMI: Body Mass Index; BRI: Body Roundness Index; CI: Confidence Interval; OR: Odds Ratio; Q1: quartile 1; Q4: quartile 4; WC: Waist Circumference; WHtR: Waist-to-Height Ratio.

**Table 3:** Subgroup meta-analysis based on type of population and HTA criteria.

		Men			Women			Total		
	Subgroup Analyses	N	AUC (95% CI)	I^2^	N	AUC (95% CI)	I^2^	N	AUC (95% CI)	I^2^
	Type of population									
BRI	Chinese population	4	0.65 (0.62–0.68)	94%	4	0.70 (0.64–0.75)	98%	5	0.67 (0.64–0.71)	98%
	Non-Chinese population	5	0.64 (0.62–0.67)	50%	5	0.66 (0.62–0.71)	48%	6	0.67 (0.64–0.70)	95%
	European population	2	0.67 (0.60–0.73)	79%	2	0.69 (0.66–0.72)	97%	3	0.71 (0.66–0.76)	97%
	HTA Criteria									
	130/85 mmHg	5	0.64 (0.62–0.67)	50%	5	0.66 (0.62–0.71)	48%	6	0.67 (0.64–0.84)	95%
	140/90 mmHg	4	0.65 (0.62–0.68)	94%	4	0.70 (0.64–0.75)	98%	5	0.67 (0.64–0.71)	98%
ABSI	Type of population									
	Chinese population	4	0.58 (0.56–0.60) ^c^	80%	4	0.61 (0.58–0.65) ^b^	96%	5	0.60 (0.58–0.62) ^c^	95%
	Non-Chinese population	5	0.54 (0.50–0.58) ^c^	84%	5	0.57 (0.54–0.61) ^c^	81%	6	0.57 (0.54–0.60) ^c^	91%
	European population	2	0.59 (0.49–0.69)	92%	2	0.58 (0.56–0.60) ^c^	78%	3	0.60 (0.55–0.65) ^c^	94%
	HTA Criteria									
	130/85 mmHg	5	0.54 (0.50–0.58) ^c^	84%	5	0.57 (0.54–0.61) ^c^	81%	6	0.57 (0.54–0.60) ^c^	91%
	140/90 mmHg	4	0.58 (0.56–0.60) ^c^	80%	4	0.61 (0.58–0.65) ^b^	96%	5	0.60 (0.58–0.62) ^c^	95%
BMI	Type of population									
	Chinese population	3	0.64 (0.61–0.68) ^‡^	92%	3	0.68 (0.62–0.75) *	98%	4	0.66 (0.62–0.69) ^†^	97%
	Non-Chinese population	4	0.67 (0.65–0.69) ^‡^	29%	4	0.64 (0.59–0.69) *	83%	5	0.67 (0.64–0.80) ^‡^	86%
	European population	2	0.68 (0.64–0.71) ^‡^	73%	2	0.68 (0.65–0.70) ^‡^	25%	3	0.69 (0.66–0.73) ^‡^	90%
	HTA Criteria									
	130/85 mmHg	4	0.67 (0.65–0.69) ^‡^	29%	4	0.64 (0.59–0.69) *	83%	5	0.67 (0.64–0.80) ^‡^	86%
	140/90 mmHg	3	0.64 (0.61–0.68) ^‡^	92%	3	0.68 (0.62–0.75) *	98%	4	0.66 (0.62–0.69) ^†^	97%
WC	Type of population									
	Chinese population	3	0.66 (0.64–0.68) ^‡^	80%	3	0.70 (0.63–0.77) ^†^	99%	4	0.68 (0.65–0.71) ^‡^	98%
	Non-Chinese population	4	0.66 (0.64–0.68) ^‡^	0%	4	0.68 (0.66–0.71) ^‡^	68%	5	0.69 (0.66–0.71) ^‡^	82%
	European population	2	0.66 (0.64–0.68) ^‡^	0%	2	0.69 (0.68–0.70) ^‡^	0%	3	0.70 (0.67–0.73) ^‡^	88%
	HTA Criteria									
	130/85 mmHg	4	0.66 (0.64–0.68) ^‡^	0%	4	0.68 (0.66–0.71) ^‡^	68%	5	0.69 (0.66–0.71) ^‡^	82%
	140/90 mmHg	3	0.66 (0.64–0.68) ^‡^	80%	3	0.70 (0.63–0.77) *	99%	4	0.68 (0.65–0.71) ^‡^	98%
WHtR	Type of population									
	Chinese population	3	0.66 (0.62–0.69) ^‡^	92%	3	0.71 (0.64–0.77) ^†^	97%	4	0.68 (0.64–0.71) ^‡^	98%
	Non-Chinese population	3	0.67 (0.63–0.70) ^‡^	29%	3	0.68 (0.64–0.72) ^‡^	83%	4	0.69 (0.66–0.72) ^‡^	85%
	European population	2	0.67 (0.63–0.72) ^‡^	62%	2	0.70 (0.68–0.71) ^‡^	28%	3	0.71 (0.67–0.74) ^‡^	86%
	HTA Criteria									
	130/85 mmHg	3	0.67 (0.63–0.70) ^‡^	29%	3	0.68 (0.64–0.72) ^‡^	83%	4	0.69 (0.66–0.72) ^‡^	85%
	140/90 mmHg	3	0.66 (0.62–0.69) ^‡^	92%	3	0.71 (0.64–0.77) *	97%	4	0.68 (0.64–0.71) ^‡^	98%

Abbreviation: AUC: Area Under Curve; ABSI: A Body Adiposity Index; BMI: Body Mass Index; BRI: Body Roundness Index; HTA: Arterial Hypertension; I^2^: Heterogeneity; N: number of studies included; WC: Waist Circumference; WHtR: Waist-to-Height Ratio. Note: Within the non-Chinese population, only European population were also analysed, in order to distinguish the possible influence of the various populations that make up the non-Chinese. Differences between BRI and ABSI, BMI, WC or WHtR: ^a^ *p* < 0.05; ^b^ *p* < 0.01; ^c^ *p* < 0.001. Differences between ABSI and BMI, WC or WHtR: * *p* < 0.05; ^†^ *p* < 0.01; ^‡^ *p* < 0.001.

**Table 4:** Pooled accuracy parameters in the prediction of hypertension.

		N	Sensitivity	Specificity	PLR	NLR	dOR	AUC-SROC
BRI	Men	4	0.62 (0.61–0.63)	0.60 (0.60–0.61)	1.54 (1.35–1.75)	0.65 (0.55–0.76)	2.37 (1.82–3.08)	0.64 (0.59–0.68)
	Women	4	0.65 (0.64–0.66)	0.65 (0.65–0.66)	1.60 (1.13–2.27)	0.60 (0.44–0.82)	2.66 (1,42–4.96)	0.62 (0.52–0.72)
	Total	4	0.63 (0.63–0.64)	0.62 (0.62–0.63)	1.57 (1.34–1.84)	0.62 (0.54–0.72)	2.50 (1.87–3.34)	0.64 (0.60–0.69)
ABSI	Men	3	0.52 (0.51–0.53)	0.51 (0.51–0.52)	1.17 (1.02–1.34)	0.86 (0.73–1.00)	1.36 (1.05–1.77) ^b^	0.55 0.49–0.60) ^b^
	Women	4	0.48 (0.47–0.49)	0.55 (0.54–0.55)	1.33 (1.16–1.53)	0.75 (0.59–0.94)	1.78 (1.28–2.46)	0.59 (0.54–0.65)
	Total	4	0.51 (0.50–0.51)	0.53 (0.52–0.53)	1.26 (1.15–1.38)	0.79 (0.71–0.89)	1.58 (1.30–1.92) ^b^	0.57 (0.53–0.61) ^b^
BMI	Men	2	0.68 (0.67–0.69)	0.54 (0.53–0.54)	1.54 (1.51–1.57)	0.56 (0.52–0.60)	2.81 (2.67–2.97) ^‡^	-
	Women	2	0.58 (0.54–0.56)	0.67 (0.66–0.67)	2.14 (1.95–2.35)	0.54 (0.35–0.83)	3.99 (2.99–5.31)	-
	Total	2	0.63 (0.62–0.63)	0.59 (0.59–0.60)	1.84 (1.50–2.25)	0.55 (0.46–0.65)	3.33 (2.57–4.3) ^‡^	0.69 (0.65–0.73) ^‡^
WC	Men	2	0.56 (0.55–0.57)	0.58 (0.58–0.59)	1.39 (1.06–1.83)	0.72 (0.45–1.16)	1.91 (0.93–3.923)	-
	Women	2	0.61 (0.60–0.62)	0.65 (0.64–0.66)	1.99 (1.93–2.04)	0.52 (0.38–0.73)	3.75 (2.75–5.12)	-
	Total	2	0.58 (0.57–0.59)	0.61 (0.61–0.62)	1.67 (1.42–1.96)	0.62 (0.48–0.79)	2.69 (1.91–3.79) ^†^	0.68 (0.63–0.74) ^†^
WHtR	Men	3	0.64 (0.63–0.64)	0.61 (0.60–0.61)	1.54 (0.30–1.82)	0.61 (0.52–0.71)	2.54 (1.95–3.31) ^‡^	0.66 (0.60–0.71) ^†^
	Women	3	0.65 (0.64–0.66)	0.69 (0.68–0.69)	1.92 (1.51–2.44)	0.56 (0.41–0.76)	3.44 (2.08–5.67)	0.72 (0.66–0.79) ^†^
	Total	3	0.64 (0.63–0.65)	0.64 (0.64–0.65)	1.71 (1.46–2.01)	0.58 (0.51–0.67)	2.94 (2.23–3.89) ^‡^	0.67 (0.61–0.72) ^†^

Abbreviation: AUC-SROC: Area Under Curve-Summary Receiver Operating Characteristic; ABSI: A Body Adiposity Index; BMI: Body Mass Index; BRI: Body Roundness Index; dOR: Diagnostic Odds Ratio; N: number of studies included; NLR: Negative Likelihood Ratio; PLR: Positive likelihood Ratio; WC: Waist Circumference; WHtR: Waist-to-Height Ratio. dOR and AUC-SROC differences between BRI and ABSI, BMI, WC or WHtR: ^a^ *p* < 0.05; ^b^ *p* < 0.01; ^c^ *p* < 0.001. dOR and AUC-SROC differences between ABSI and BMI, WC or WHtR: * *p* < 0.05; ^†^ *p* < 0.01; ^‡^ *p* < 0.001.

## Data Availability

Not applicable.

## Supplementary Materials

The following are available online at https://www.mdpi.com/article/10.3390/ijerph182111607/s1, Table S1. Quality assessment of studies included in the review; Table S2. Baseline sensitivity and specificity data of included studies; Figure S1. Random effect meta-regression model in the total population for BRI (A), ABSI (B), BMI (C), WC (D), and WHtR (E); Figure S2. Random effect meta-regression model in men for BRI (A), ABSI (B), BMI (C), WC (D), and WHtR (E); Figure S3. Random effect meta-regression model in women for BRI (A), ABSI (B), BMI (C), WC (D), and WHtR (E); Figure S4. Estimated pooled AUC-SROC in the total population for BRI (A), ABSI (B), BMI (C), WC (D), WHtR (E); Figure S5. Estimated pooled AUC-SROC in men for BRI (A), ABSI (B), and WHtR (C); Figure S6. Estimated pooled AUC-SROC in women for BRI (A), ABSI (B), and WHtR (C).
