# Supplementary material for: Effectiveness of Body Roundness Index (BRI) and a Body Shape Index (ABSI) in Predicting Hypertension: A Systematic Review and Meta-Analysis of Observational Studies
Source: Int J Environ Res Public Health. 2021 Nov 4;18(21):11607. doi: 10.3390/ijerph182111607 (PMC8582804; doi:10.3390/ijerph182111607)
Supplement: Supplementary file 1 [file ijerph-18-11607-s001.zip › ijerph-1408412-supplementary.pdf]

Table S1. Quality assessment of studies included in the review

Study ID criteria	1	2	3	4	5	6	7	8	9	10	11	12	13
1. Was the research question or objective in this paper clearly stated?	Y	Y	Y	Y	Y	Y	Y	Y	Y	Y	Y	Y	y
2. Was the study population clearly specified and defined?	Y	Y	Y	Y	Y	Y	Y	Y	Y	Y	Y	Y	Y
3. Was the participation rate of eligible persons at least 50%?	Y	Y	Y	Y	N	Y	Y	Y	Y	Y	Y	Y	Y
4. Were the all the subjects selected or recruited from the same or similar population (including the same time period? Were inclusion and exclusion criteria for being in the study pre-specified and applied uniformly to all participants?	Y	Y	Y	Y	Y	Y	Y	Y	Y	Y	Y	Y	Y
5. Was a simple size justification, power description or variance and effect estimates provided?	N	N	N	N	N	N	N	Y	N	N	N	N	N
6. For the analysis in this paper, were the exposure(s) of interest measured prior to the outcome(s) being measured?	N	N	N	N	Y	N	N	N	N	N	N	N	N
7. Was the timeframe sufficient so that one could reasonably expect to see an association between exposure and outcome if it existed?	N	N	N	N	Y	N	N	N	N	N	N	N	N
8. For exposures that can vary in amount or level, did the study examine different levels of the exposure as related to the outcome (e.g categories of exposure or exposure measured as continuous variable)?	Y	Y	Y	Y	Y	Y	Y	Y	Y	Y	Y	Y	Y
9. Were the exposure measures (indepent variable) clearly defined, valid, reliable and implemented consistently across all study participant?	Y	Y	Y	Y	Y	Y	Y	Y	Y	Y	Y	Y	Y
10. Was the exposure(s) assessed more than once over time?	N	N	N	N	Y	N	N	N	N	N	N	N	N
11. Were the outcome measures (dependent variables) clearly defined, valid reliable and implemented consistently across all study participants?	Y	Y	Y	Y	Y	Y	Y	Y	Y	Y	Y	Y	Y
12. Were the outcome assessors blinded to the exposure status of participants?	Y	Y	Y	Y	Y	Y	Y	Y	Y	Y	Y	Y	Y
13. Was loss to follow-up after baseline 20% or less?	CD	CD	CD	CD	Y	CD	CD	CD	CD	CD	CD	CD	CD
14. Were key potential confounding variables measured and adjusted statistically for their impact relationship between exposure(s) and outcome(s)?	N	Y	N	Y	Y	N	N	N	Y	Y	Y	N	Y
Sum of SCOREs	8	9	8	9	12	8	8	9	9	9	9	8	9
Abbreviations: CD: cannot determine, N: no; Y: Yes													

Table S2. Baseline sensitivity and specificity data of included studies.

		MEN							WOMEN						
	**Study**	**Cutt-off**	**Sen**	**Spe**	**TP**	**TN**	**FN**	**FP**	**Cutt-off**	**Sen**	**Spe**	**TP**	**TN**	**FN**	**FP**
**BRI**	Baveicy K 2020	4.690	0.636	0.545	380	2177	217	1817	6.050	0.637	0.501	495	1713	282	1709
	Gluszek S 2020	4.855	0.538	0.677	1814	489	1558	233	4.931	0.635	0.566	3660	1398	2104	1072
	Liu PJ 2017	2.800	0.545	0.600	163	247	136	165	2.600	0.542	0.645	104	447	88	246
	Zhang J 2018	3.810	0.665	0.614	4913	17628	2475	11082	3,330	0.717	0.694	1994	13984	787	6166
**ABSI**	Baveicy K 2020	0.1300	0.602	0.425	359	1697	238	2297	0.1400	0.557	0.500	433	1711	344	1711
	Gluszek S 2020	0.0830	0.346	0.715	1167	516	2205	206	0.0780	0.383	0.727	2208	1796	3556	674
	Liu PJ 2017	-	-	-	-	-	-	-	0.0742	0.589	0.577	113	400	79	293
	Zhang J 2018	0.079	0.604	0.523	4462	15015	2926	13695	0.0758	0.682	0.537	1897	10821	884	9329
**BMI**	Baveicy K 2020	-	-	-	-	-	-	-	-	-	-	-	-	-	-
	Gluszek S 2020	27.18	0.651	0.594	2195	429	1177	293	28.44	0.488	0.783	2813	1934	2951	536
	Liu PJ 2017	-	-	-	-	-	-	-	-	-	-	-	-	-	-
	Zhang J 2018	24.75	0.705	0.542	5209	15561	2179	13149	23.18	0.704	0.663	1958	13359	823	6791
**WC**	Baveicy K 2020	-	-	-	-	-	-	-	-	-	-	-	-	-	-
	Gluszek S 2020	98.00	0.346	0.715	1167	516	2205	206	88.00	0.564	0.712	3251	1759	2513	711
	Liu PJ 2017	-	-	-	-	-	-	-	-	-	-	-	-	-	-
	Zhang J 2018	82.25	0.662	0.584	4891	16767	2497	11943	78.20	0.706	0.647	1963	13037	818	7113
**WHtR**	Baveicy K 2020	-	-	-	-	-	-	-	-	-	-	-	-	-	-
	Gluszek S 2020	0.56	0.582	0.647	1963	467	1409	255	0.54	0.631	0.649	3637	1603	2127	867
	Liu PJ 2017	0.47	0.681	0.459	204	189	95	223	0.47	0.438	0.733	84	508	108	185
	Zhang J 2018	0.52	0.665	0.614	4913	17628	2475	11082	0.50	0.715	0.694	1988	13984	793	6166

Abbreviation: ABSI: A Body Shape Index; BMI: Body Mass Index; BRI: Body Roundness Index; FN: False negative value; FP: False positive value ; Sen; Sensitivity; Spe: Specificity; TP: True positive value; TN: True negative value; WC: Waist circumference; WHtR: Waist-to-Height Ratio.

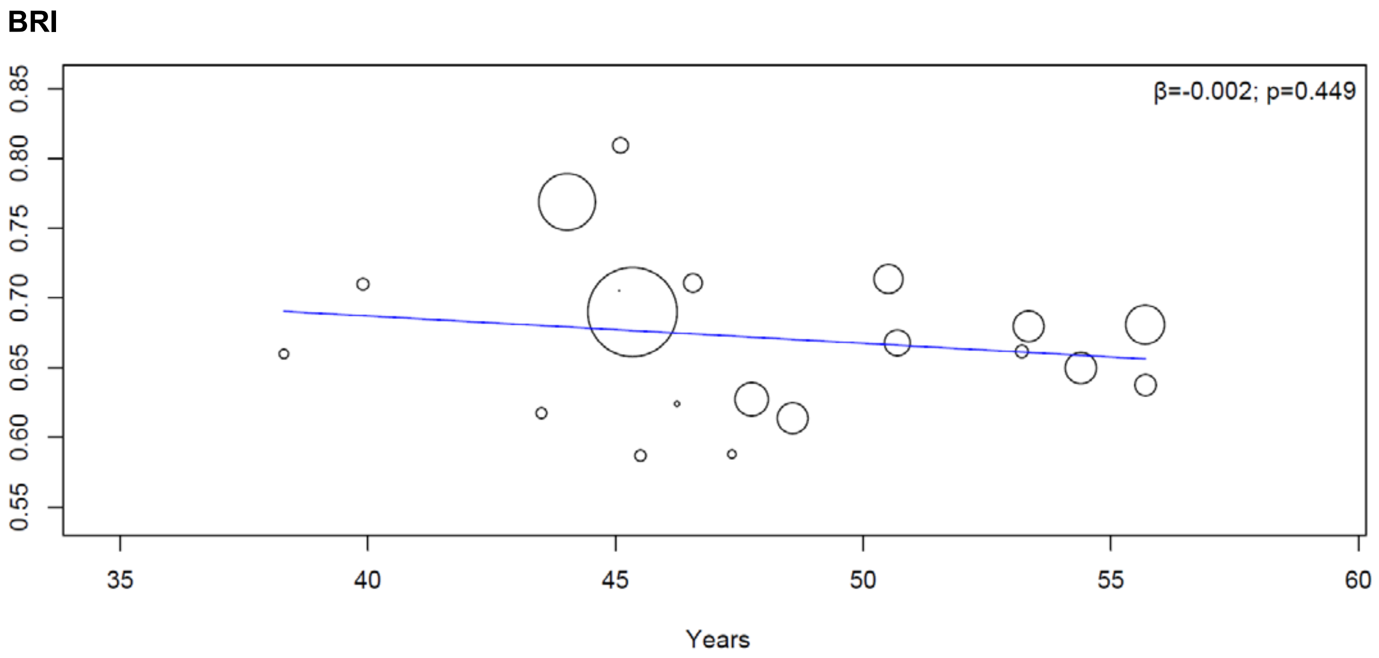

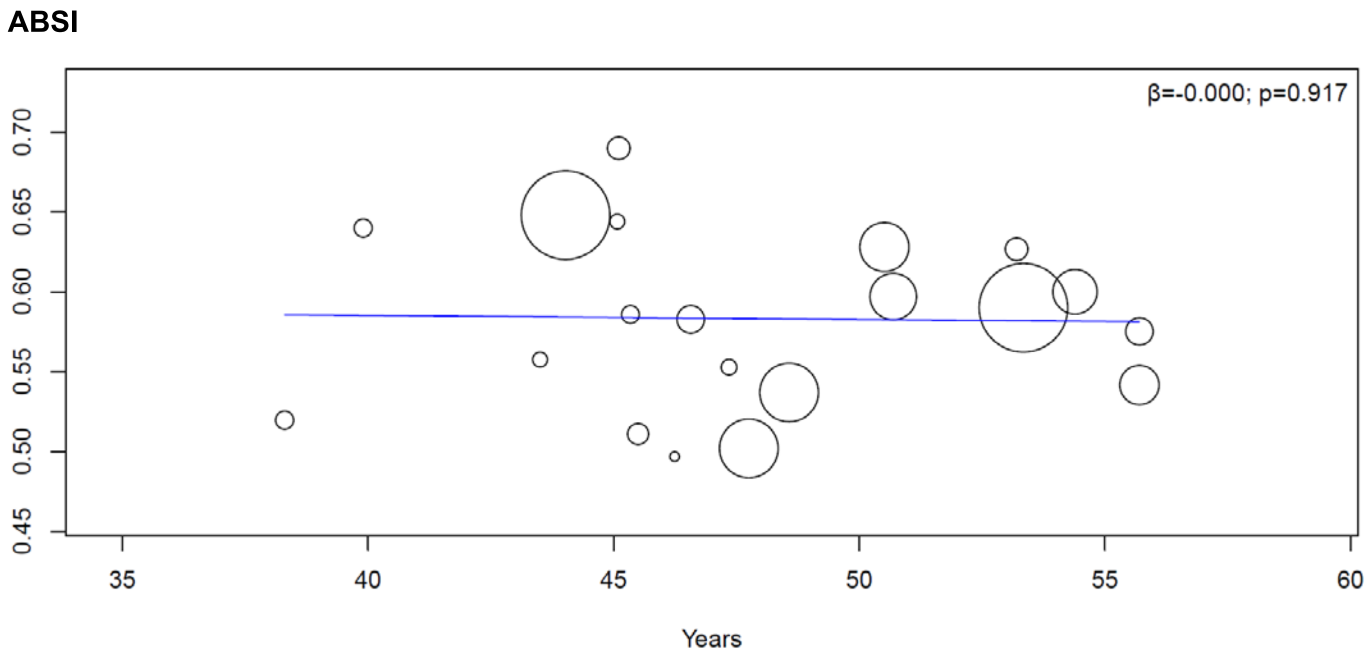

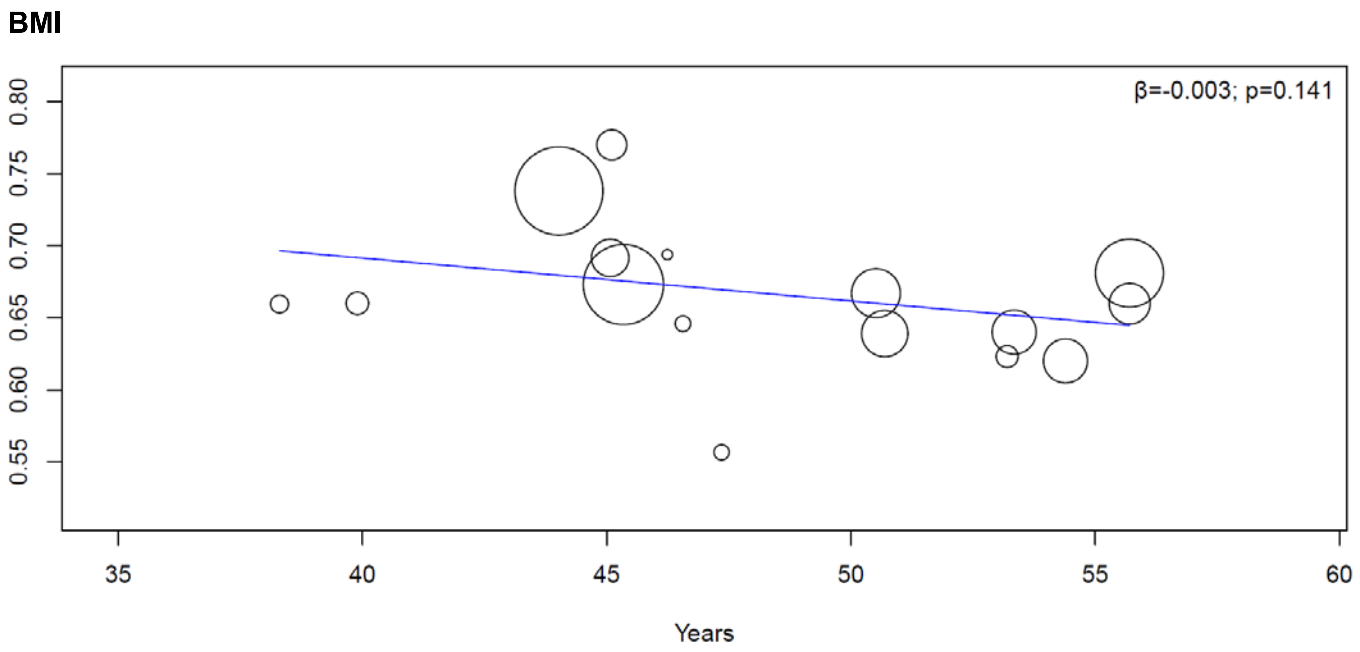

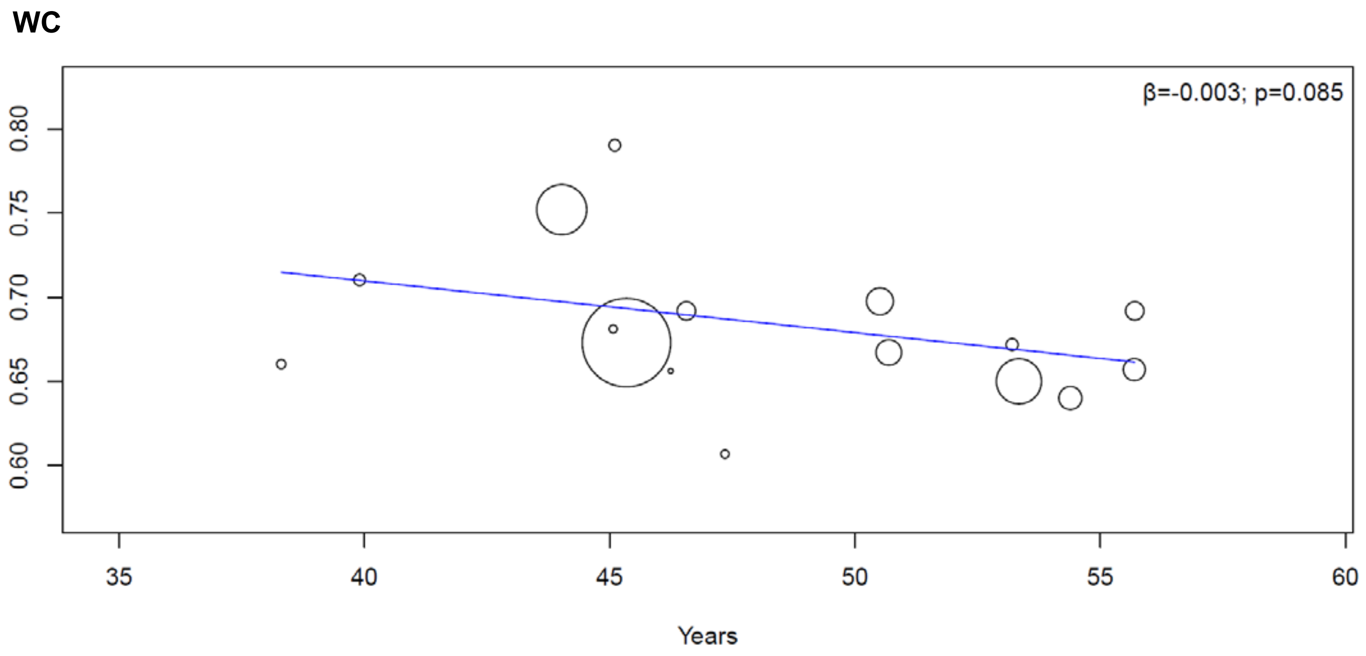

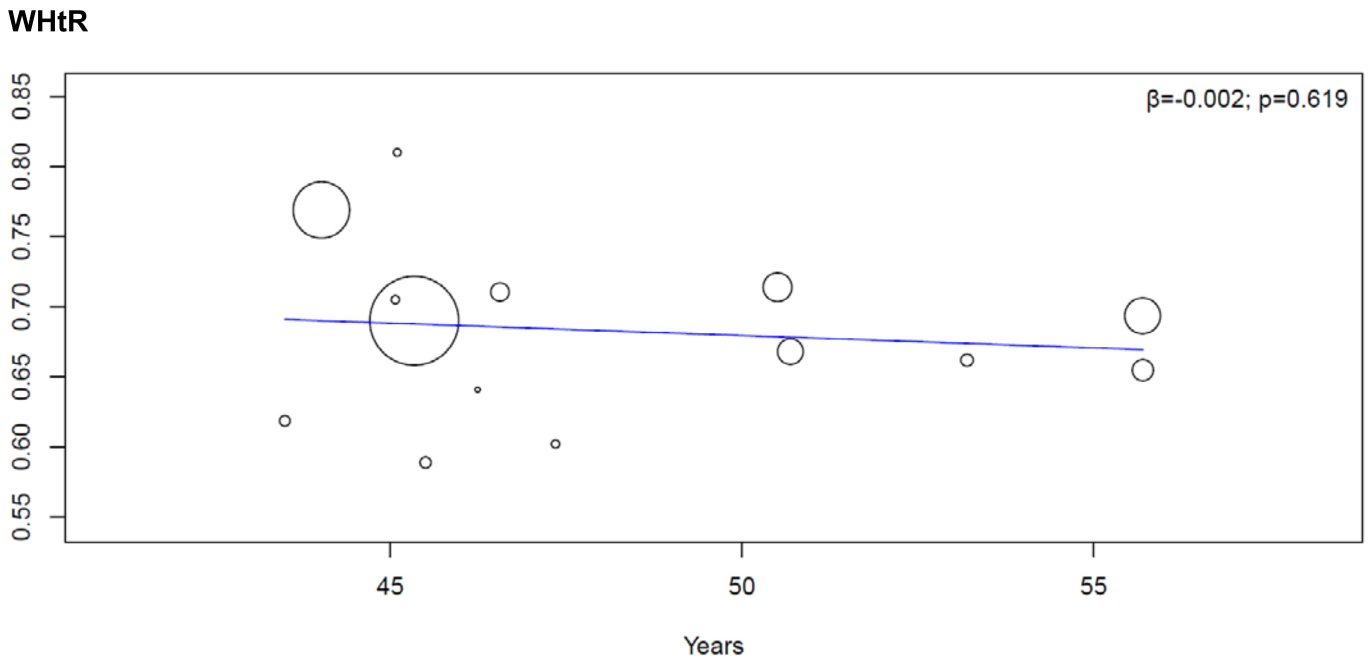

Figure S1. Random effect meta-regression model in the total population for BRI, ABSI, BMI, WC and WHtR.

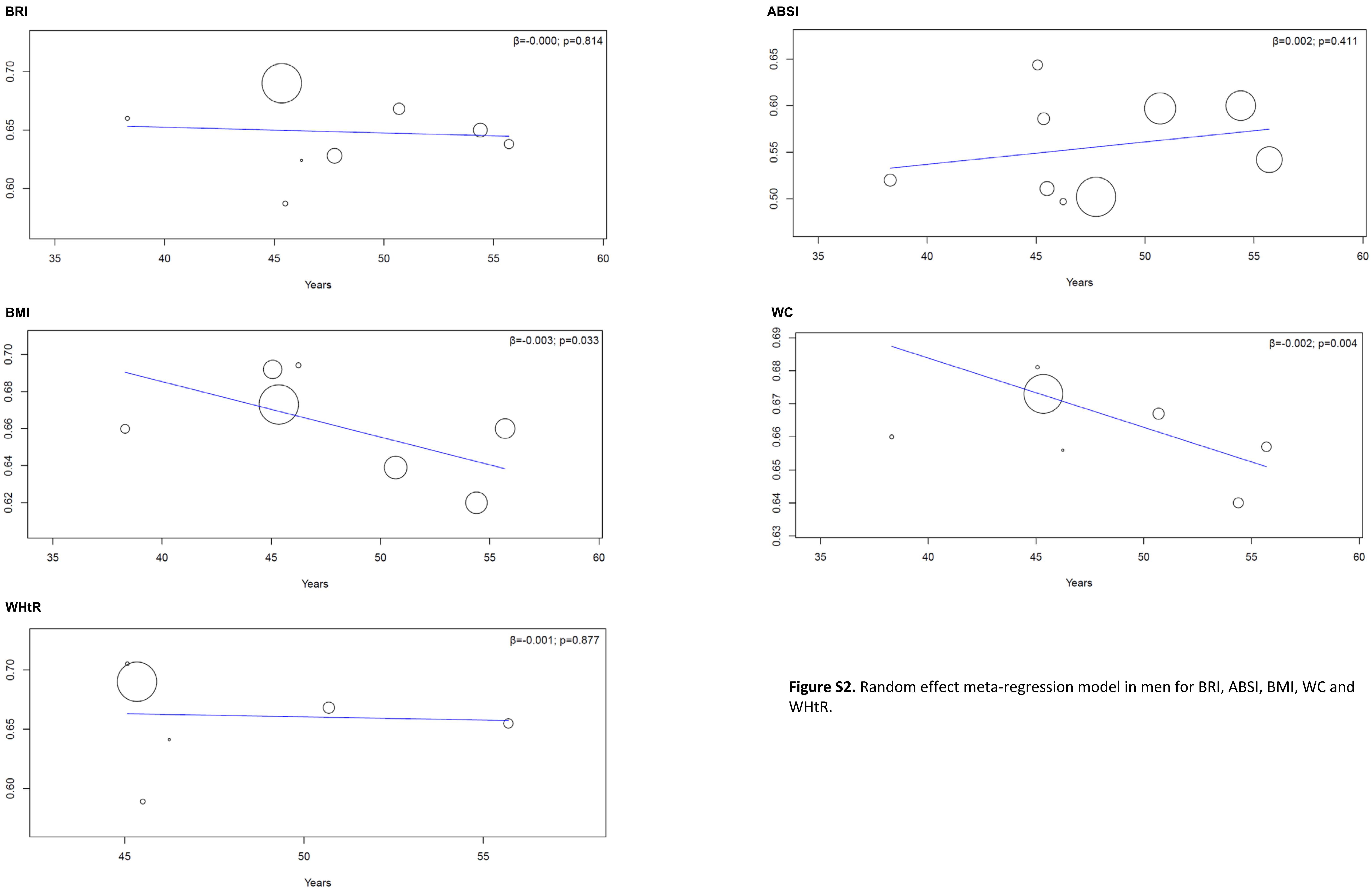

Figure S2. Random effect meta-regression model in men for BRI, ABSI, BMI, WC and WHtR.

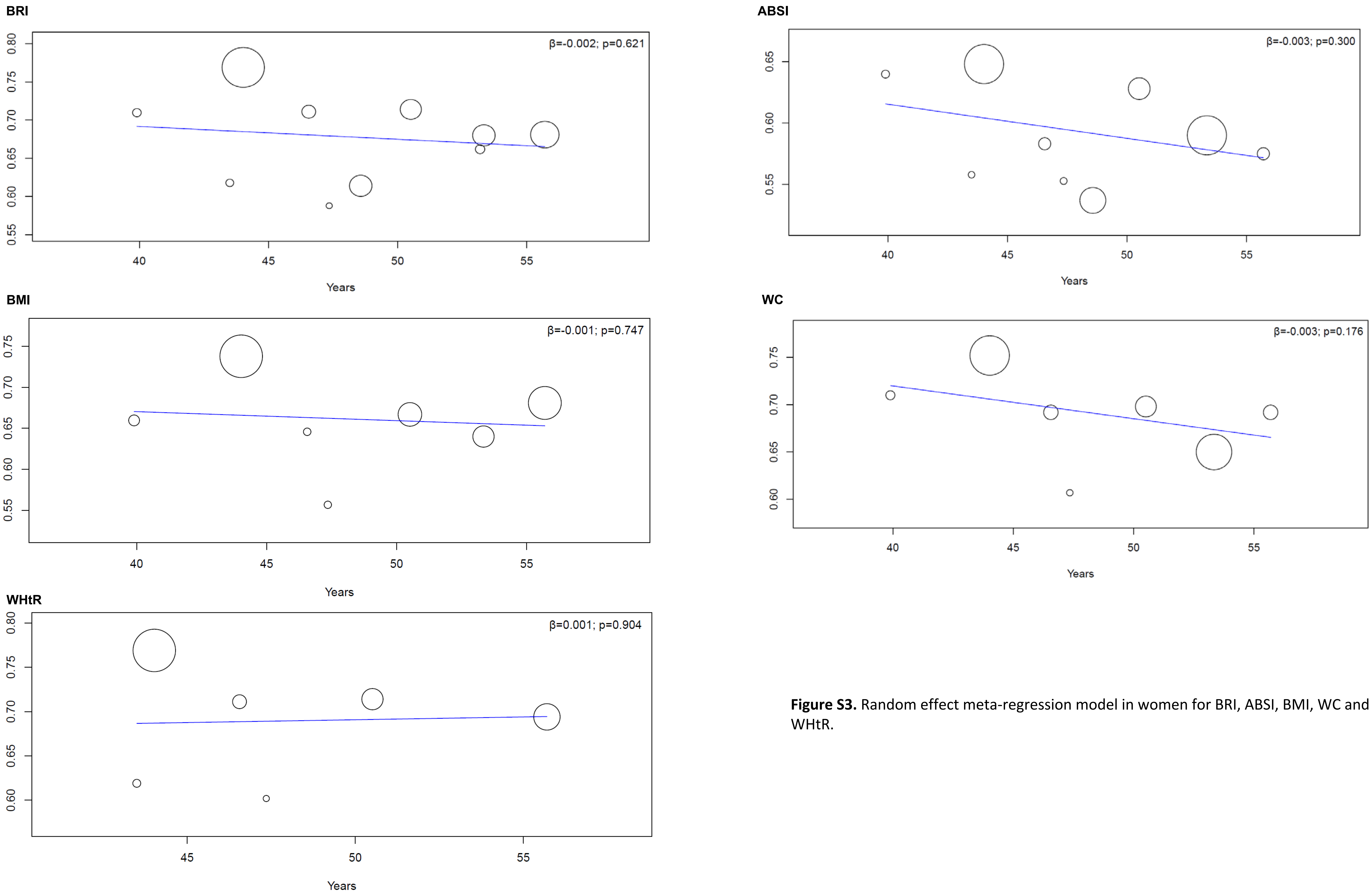

Figure S3. Random effect meta-regression model in women for BRI, ABSI, BMI, WC and WHtR.

BRI

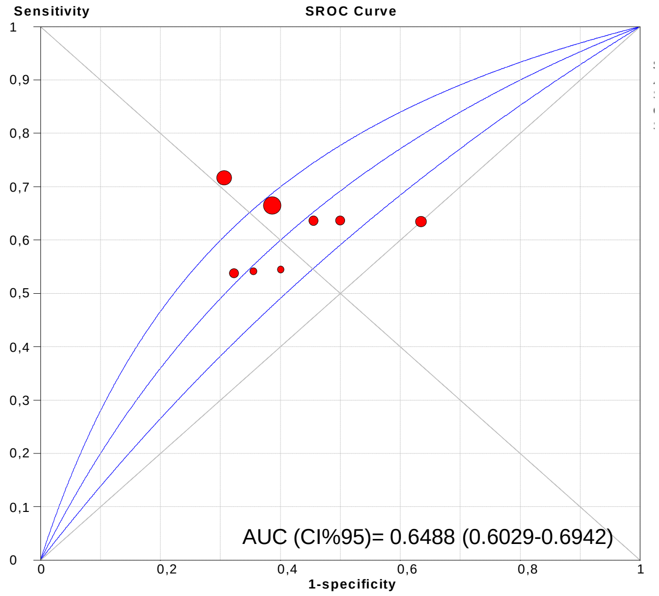

ABSI

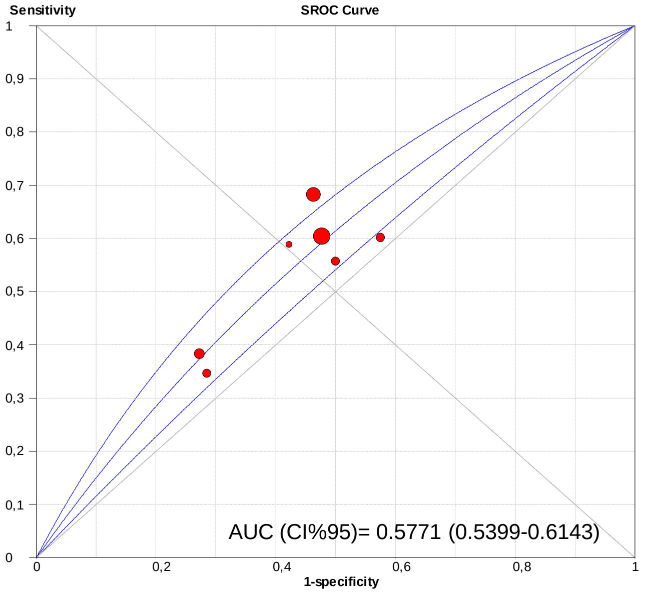

BMI

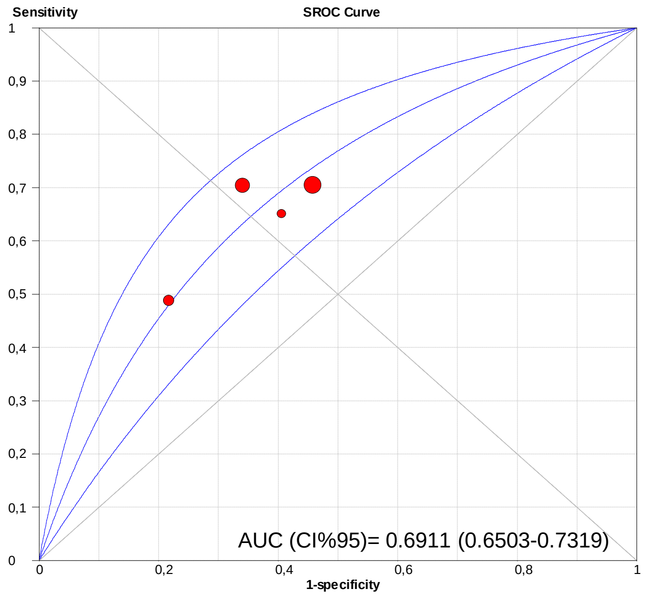

WC

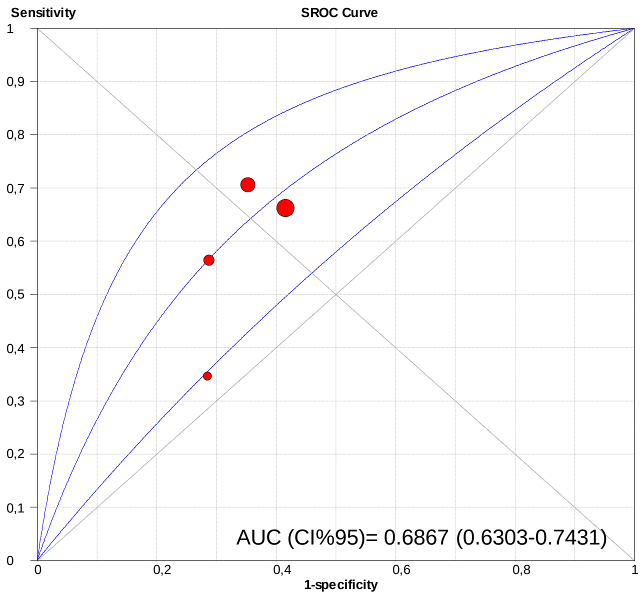

WHtR

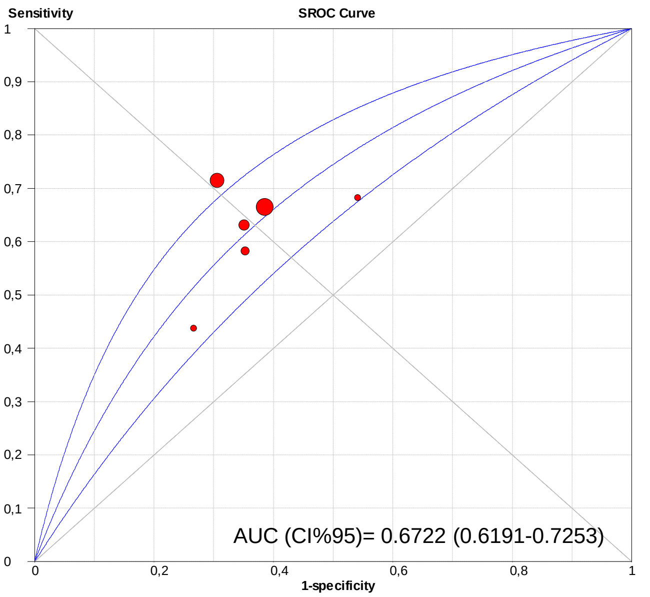

Figure S4. Estimated pooled AUC-SROC in the total population for BRI, ABSI, BMI, WC and WHtR.

BRI

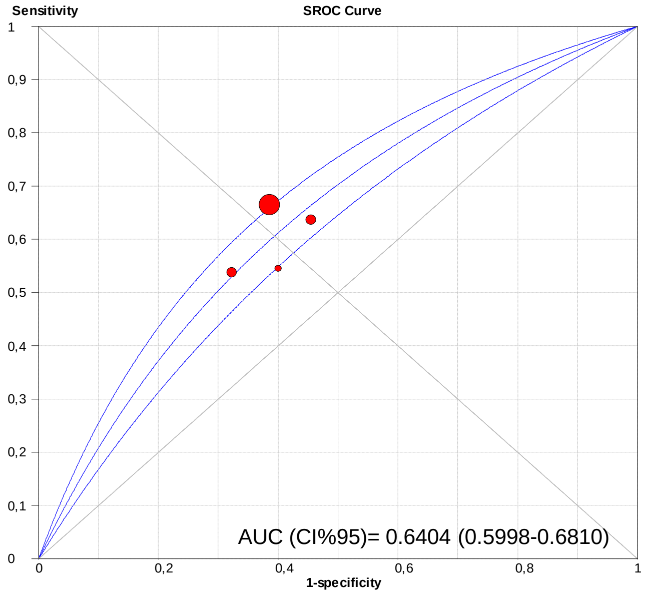

ABSI

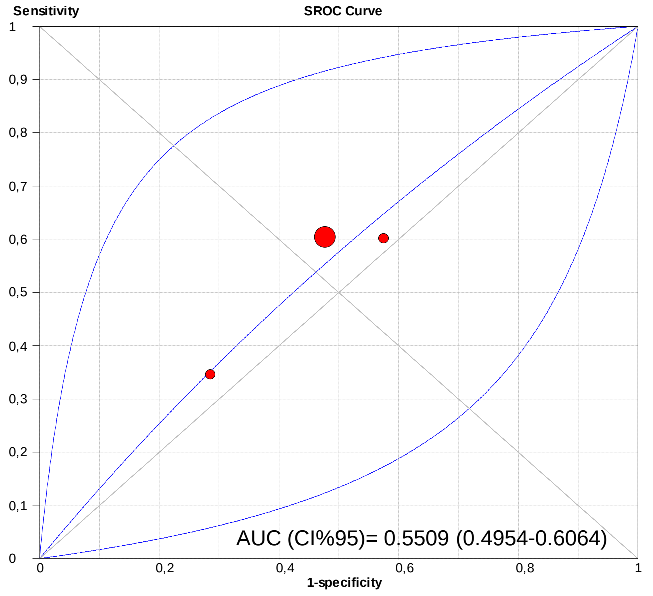

WHtR

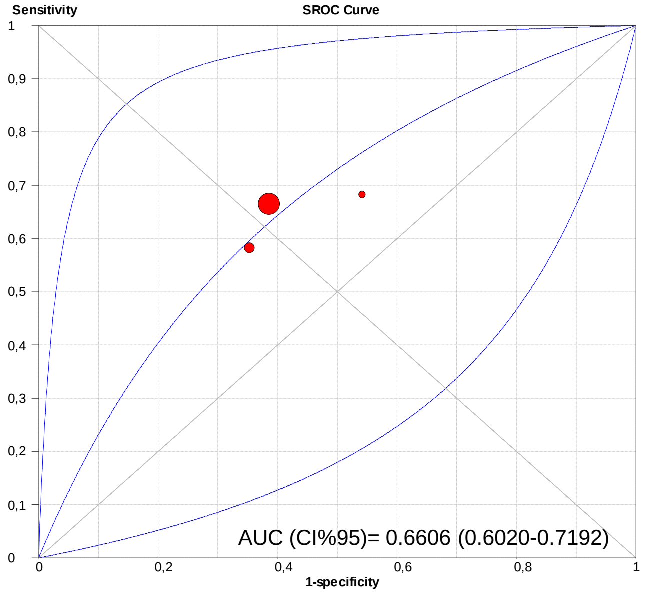

Figure S5. Estimated pooled AUC-SROC in men for BRI, ABSI and WHtR.

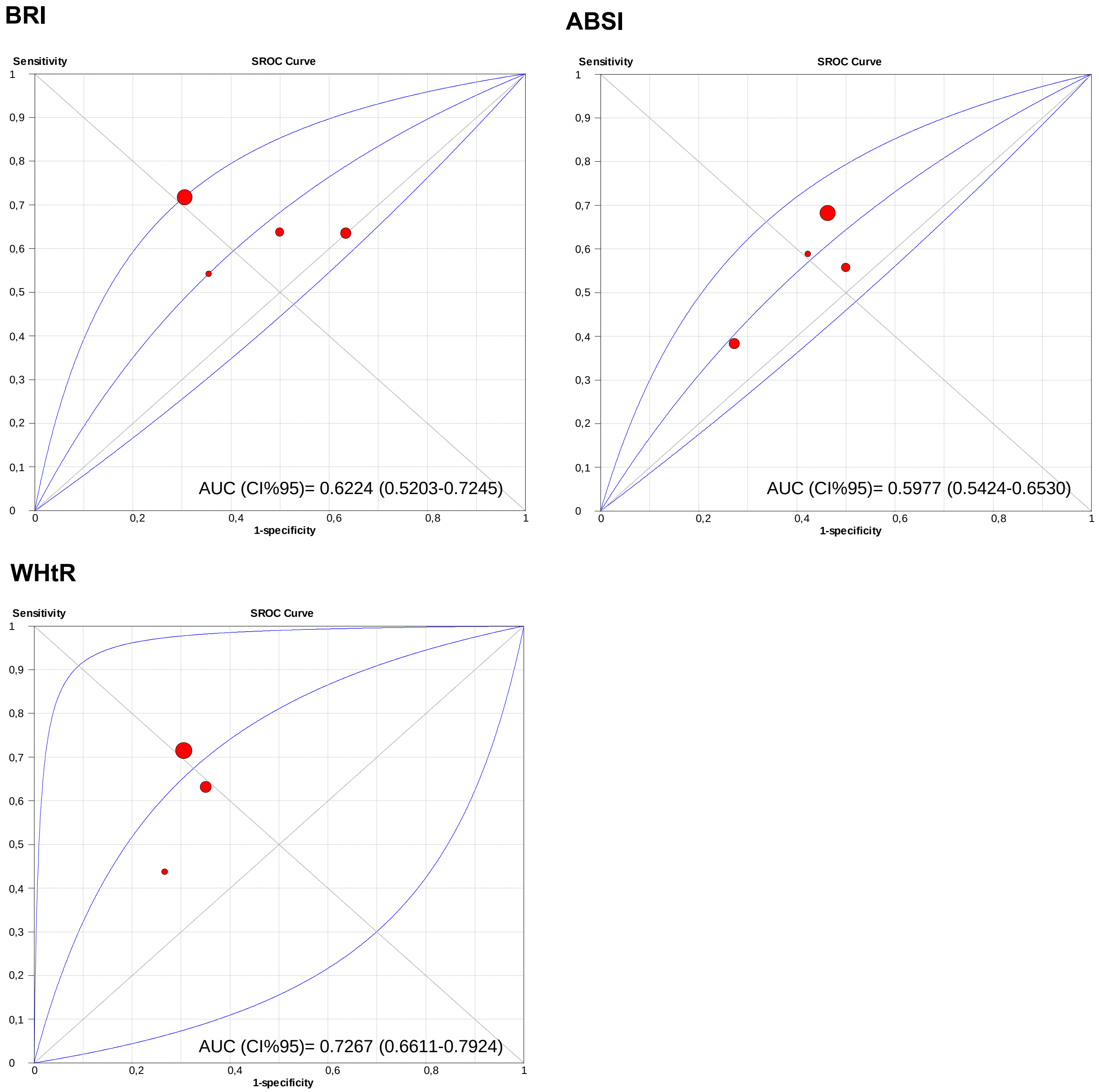

Figure S6. Estimated pooled AUC-SROC in women for for BRI, ABSI and WHtR.
